# Investigating the balance between goal-directed and habitual control in experimental and real-life settings

**DOI:** 10.3758/s13420-018-0313-6

**Published:** 2018-02-09

**Authors:** Floris E. Linnebank, Merel Kindt, Sanne de Wit

**Affiliations:** Department of Clinical Psychology, Amsterdam Brain and Cognition Center, University of Amsterdam, Nieuwe Achtergracht 129-B, 1087 AW Amsterdam, Netherlands

**Keywords:** Habit propensity, Action control, Individual differences, Dual-system theory

## Abstract

Do people differ in their propensity to form habits? The current study related individual differences in habitual performance on the slips-of-action task to habit formation in real life. To this end, we developed a novel key-cover procedure that controls for the amount of repetition and motivation within a naturalistic setting. Participants received a key cover for the key to their home, which after several weeks was switched with a key cover that was previously attached to a dummy key. Participants recorded effort, time, attention, and mistakes in the key-selection process. Results were in line with established properties of habits, as attention decreased in the learning phase, yet effort, time, and mistakes increased after the key-cover switch. Performance on the slips-of-action task correlated negatively with changes in attention in the real-life key-cover task. This negative correlation may reflect that flexible behavioral adjustment requires more attention in people with a relatively weak goal-directed system.

**Electronic supplementary material:**

The online version of this article (10.3758/s13420-018-0313-6) contains supplementary material, which is available to authorized users.

Could it be advantageous to quickly form strong habits? As an example of habit formation, consider an ordinary everyday behavior such as opening the door to your home. While multiple keys may be on your key set, the correct key must be selected and inserted into the lock. Initially this involves an effortful, conscious process with the goal of opening the front door clearly in mind, but with some repetition, this behavior can become habitual. Indeed, self-report research in naturalistic settings suggests that repeated goal pursuit in stable contexts leads to habit formation (Neal, Wood, & Quinn, 2006; Ouellette & Wood, 1998; Wood & Neal, 2007). Habits may play an important role in everyday life, as it is estimated that almost half of our daily behavior is performed repetitively in stable contexts (Wood, Quinn, & Kashy, 2002). Once formed, such habits are associated with efficiency and lower awareness (Bargh, 1994; Wood et al., 2002). Therefore, with practice, opening the door should be achieved in less time, with less effort, and whilst requiring less attention.

Even though habits are generally very functional, habits may not always be beneficial. Their downside may be inflexibility, as habits are also characterized by rigidity, an invariable response (Wood & Neal, 2007), and a lack of control (Bargh, 1994). To illustrate, in our example, when keys or locks are changed, we may find ourselves perseverating in trying to open the door with the wrong (old) key.

Dual-system accounts of action control attribute this gradual loss of flexibility with repetition to a shift in the balance between two systems (e.g., de Wit & Dickinson, 2009; Dickinson, 1985; Dolan & Dayan, 2013). The goal-directed system determines what response is favorable on the basis of (1) knowledge of response–outcome contingencies, and (2) evaluation of the current desirability of each outcome. The concurrently operating habit system, however, drives behavior through simple stimulus–response associations that are strengthened by repetition. Initially, the goal-directed system will exert dominant control, but when a behavior is repeatedly performed in a stable context, the stimulus–response associations in the habit system will become sufficiently strong to drive behavior directly. As a consequence, when a certain response no longer leads to a desirable or valued outcome, it may still be executed due to strong input from the habit system. This has been shown most extensively in animal research (for the original demonstration, see Adams, 1982), and more recently, also in humans (Tricomi, Balleine, & O’Doherty, 2009). Furthermore, animal lesion studies of habit formation as well as human neuroimaging studies have related goal-directed and habitual control to two dissociable corticostriatal neural systems (Balleine & O’Doherty, 2010; Liljeholm, Tricomi, O’Doherty, & Balleine, 2011; Smittenaar, FitzGerald, Romei, Wright, & Dolan, 2013; Tanaka, Balleine, & O’Doherty, 2008; Valentin, Dickinson, & O’Doherty, 2007).

Behavioral inflexibility—as reflected, for example, in “slips of action,” such as trying to open a door with an obsolete key—is not merely a result of behavioral repetition. Such a relative dominance of the habit system can also be induced by stress (Schwabe & Wolf, 2009, 2011) and fatigue, as the latter proposedly decreases self-control resources (Muraven, Tice, & Baumeister, 1998). The question arises whether there are individual differences in the inherent strength of either system, thus influencing the balance independently of the abovementioned variables and thus leading to relatively faster habit formation or stronger habit expression. May some individuals therefore be *generally* more prone than others to act out of habit? In other words, do people differ in their habit propensity, some being more a creature of habit than others?

The notion of habit propensity was first introduced to account for behavioral inflexibility and a seeming loss of control in the pathogenesis of several psychiatric disorders that involve impulsive-compulsive behavior (Robbins, Gillan, Smith, de Wit, & Ersche, 2012). Initial evidence for this idea has been provided by studies that have employed the slips-of-action paradigm. To briefly explain this instrumental-learning task: an initial training phase on different stimulus–response–outcome contingencies is followed by a devaluation of some of the outcomes. Participants are tested to see if they can refrain from performing learnt responses to the stimuli associated to these devalued outcomes while still responding quickly to the stimuli associated with still-valued outcomes. Their performance on this test is taken as a measure of the balance between goal-directed and stimulus-response habitual control. Individuals with obsessive-compulsive disorder have been shown to perform relatively habitual on this task (Gillan et al., 2011; but also with a shock-avoidance paradigm, see Gillan, Morein-Zamir, Urcelay, et al., 2014a; with an economic-choice paradigm, see Gillan, Morein-Zamir, Kaser, et al., 2014b, and with a two-step paradigm, see Voon et al., 2014). Similarly, performance of Tourette syndrome patients (Delorme et al., 2016) and cocaine users was impaired relative to healthy controls (Ersche et al., 2016). Other studies, using a very similar task, have provided initial evidence for stronger “habit propensity” in individuals with alcohol dependence (Sjoerds et al., 2013) and Parkinson’s disease (de Wit, Barker, Dickinson, & Cools, 2011(de Wit et al. 2011)). Furthermore, individual differences amongst subclinical participants in impulsivity (as measured with the Barrett Impulsiveness Scale (Patton, Standord, & Barratt, 1995) and obsessive-compulsive symptoms (as measured with the Obsessive-Compulsive Inventory [OCI]; Foa et al., 2002) have also been related to insensitivity to outcome devaluation (Hogarth, Chase, & Baess, 2012, Snorrason, Lee, de Wit, & Woods, 2016). Perhaps most convincingly, a recent study suggests that individual differences in the balance between goal-directed and habitual control have a structural neurological basis. In this study, performance on the slips-of-action task was associated with white-matter connectivity in the associated corticostriatal circuits (de Wit, Watson, et al., 2012a; see also Delorme et al., 2016). Finally, while neuroimaging research in humans can only provide correlational support for dissociable corticostriatal circuitries controlling goal-directed and habitual behavior, animal lesioning research provides converging evidence, with lesions to the prelimbic cortex and dorsomedial striatum leading to habitual behavior (Balleine & O’Doherty, 2010). Drug exposure has also been shown to lead to habit propensity in animals (Nelson & Killcross, 2006).

To summarize, converging evidence from experimental research suggests that people may differ in their disposition towards habit learning. This “habit propensity” could be clinically relevant factor, whether it be as a “temperamental” preexisting vulnerability or as an acquired maintaining factor. However, the notion of habit propensity is still far from being established as a relatively stable personal characteristic, although related differences in brain structure provide a compelling argument. To draw such a conclusion, research is needed on temporal stability as well as on effects across environments and tasks. The present study focusses on the latter aspect and therefore sets out to investigate differences in habit propensity in real-life settings and how such everyday habitual behavior relates to experimental measurements of habit propensity.

To investigate habit propensity, external factors influencing the extent of habit formation and expression need to be controlled. First of all, the amount of repetition must be controlled. This means that behavior novelty must be controlled, thus ensuring that automaticity of the target behavior is equal for all participants at the start of the study. Furthermore, a stable cue and context for the target behavior is needed (Judah, Gardner, & Aunger, 2013; Lally & Gardner, 2013), and the behavior should be performed relatively frequently during the study, as the time before a habit reaches its maximal self-reported strength can vary widely, from a couple of weeks to more than half a year (Lally, Van Jaarsveld, Potts, & Wardle, 2010). Finally, motivational influences must be controlled, as they are likely to influence goal-directedness (e.g., Gardner & Lally, 2013; Hogarth, 2011). Accordingly, motivation—along with previous behavioral repetition—has predicted the rate with which habits are formed in naturalistic studies (e.g., Danner, Aarts, & de Vries, 2008; Judah et al., 2013).

To provide sufficient control over the abovementioned factors in a real-life setting, we devised a novel key-cover procedure for the target behavior of selecting the key to one’s front door. In this procedure, the habit to select a particular key cover was first established, after which this habit was disrupted by switching this key cover with a different key cover placed on a dummy key. A diary-study approach was taken to record several measures of automaticity (mistakes/effort/time/attention; see Method section) immediately after the behavior had occurred. It was hypothesized that individual differences in habit behavior displayed in real-life would be predicted by performance on the experimental slips-of-action task.

## Method

### Overall study design

The key-cover procedure was designed to measure speed of habit formation and strength of habit expression (habit propensity) in a real-life setting. First, in a 3-week (short) or 6-week (long) learning phase, a (novel) key cover was used on the home key, next to a dummy key with a another (novel) key cover of a different color. In the first (start-of-learning) and last 4 days (end-of-learning) of this phase, indicators of automaticity were measured using a diary form completed directly after every home entry. Then the key covers were switched for a 4-day test phase, in which the diary form was again completed after every home entry (switched). A two-group (short/long) design was used to explore the amount of time that is needed for habit formation to occur in this setting. In between the learning and test stages, participants completed the experimental slips-of-action task as well as a stop-signal task (included to control for response inhibition capacity).

### Participants

Participants were recruited via the university’s research website and via word of mouth. Inclusion criteria were: an age between 18 and 50 years and having a suitable front-door home key (meaning that it would accommodate a generic key cover) that was used at least daily on average. Exclusion criteria were: colorblindness, 4-day absence from home during the study period, excessive alcohol use (four or more units during more than 21 days/month), or the use of drugs on more than 1 day per week on average (given previous demonstrations that alcohol/drug dependence is associated with habit propensity; Ersche et al., 2016; Sjoerds et al., 2013), the current use of a psychoactive medication, and having been in treatment with a psychiatrist. Participants were awarded research credits or 30 euros compensation. Additionally, participants had the chance to win a pair of movie tickets in a weighted lottery based on their slips-of-action-task scores. Participants were pseudorandomly assigned to the short and long learning phase of the key-cover procedure. For further participant characteristics, see the Results section and online Supplemental Table S1.

### Materials

#### Key covers and dummy key

Participants were provided with two distinctly colored key covers, which were to be put on their home key and on a provided dummy key of similar shape. Key-cover colors were counterbalanced. If a participant already had key covers in use, distinct colors were always used. A key ring was provided to keep both keys together yet separate from other keys on the key set of the participant, to minimize the possible confounding effects of the amount of keys on the key set.

#### Home-entry diary

Indicators of automaticity during key use, as well as several state variables, were measured using a diary form. Diary entries were completed on a personal digital assistant (PDA; Palm TX), which was provided to each participant. The diary form was constructed using Pendragon Forms 5.1. Visual analogue scales (VAS) were used in which the continuous line was 48 millimeters long and had an underlying resolution of 10 points. Each VAS therefore yielded scores between zero and 10, higher scores indicating a greater support of the measured construct.

The first three items on the diary form concerned state control variables. First, as measures of fatigue, the amount of sleep during the preceding night in (half) hours was reported, and tiredness was gauged using a VAS with the cue: “At this moment I’m feeling,”, and with the anchors “tired” and “rested.” Then, stress was measured, also using a VAS with the same cue, but with the anchors: “stressed” and “relaxed.” The last four items on the form concerned automaticity indicators and are described in Table 1. The mistakes item was used as a measure of accuracy in the learning phase and as a measure of (in)flexibility in the test phase. The effort and time items were both used to measure efficiency. The final attention item was used to gauge to what extent the focus of attention was on the act of selecting the correct key to open the door.

**Table 1:** Automaticity measures in the home-entry diary used during the key-cover procedure

Item	Type	Cue	Options/Anchors	Score
Mistakes	Likert	“When I opened the door, the first thing I did was”	“grab correct key”	0
			“hesitate about dummy key	1
			“grab dummy key”	2
			“insert dummy key in lock”	3
Effort	VAS	“Selecting the right key was”	“easy” → “hard”	0–10
Time	VAS	“Selecting the right key was”	“fast” → “slow”	0–10
Attention	VAS	“When I opened the door, my thoughts were with”	“something else” → “the keys”	0–10

*Note.* For the mistakes item, participants had to indicate what their first action was when they were about to open their door. A score of 3 means that the first thing the participant did was to accidentally insert the dummy key in the lock. A score of 2 means that the participant grabbed the dummy key but refrained from going as far as inserting it into the lock (possibly because participant realized in time she or he was about to make a mistake). A score of 1 means the participant merely hesitated, but did not go as far as actually grabbing the dummy key. A score of zero means that the participant immediately grabbed the correct key

#### Slips-of-action task

This instrumental learning paradigm aims to measure the balance between goal-directed and habitual action control. The task is programmed in Visual Basic 6.0. A brief introduction is given below to introduce the reader to the task. For a detailed description, see Gillan et al., (2011), as they used a nearly identical task, the main differences being that in the current study only two test stages were used and that we used only standard instrumental discriminations (as in Worbe, Savulich, de Wit, Fernandez-Egea, & Robbins, 2015). Written as well as verbal instructions were given, which stressed the importance of learning which outcome followed each response. Instruction included a demo task question, and if participants initially answered incorrectly, the example was explained until participants understood the task. Before each phase, relevant instructions were repeated.

The task started with an instrumental discrimination training. Participants learned—by trial and error—to open boxes with different fruit images on them (stimulus), using left or right button presses (response). Correct responses resulted in rewards, consisting of different fruit images for each box (outcome) and points. No reward was given if the incorrect button was pressed. Faster correct responses within the 2-second response window led to more points being awarded. A total of six different stimuli and six different outcomes was used. Stimulus–response associations (e.g., banana-labeled box can be opened with left button) and stimulus–outcome associations (e.g., banana-labeled box has apples inside as the outcome) were counterbalanced across participants. The instrumental discrimination training consisted of six blocks of 12 trials each. Within blocks, each stimulus appeared twice in randomized order.

Subsequently, the slips-of-action test and baseline test were administered in a counterbalanced order. In the slips-of-action test, participants were instructed to respond as quickly as possible to stimuli with a still-valued outcome thereby gaining points, but to refrain from responding to stimuli with a devalued outcome as this would cost points. A 10-second screen preceding each block of trials indicated which two of the six outcomes were devalued by marking these with a superimposed red cross. The test was performed in “nominal extinction,” meaning that neither outcomes nor points earned or lost were shown. Participants were instructed, however, that their final score would be shown at the end of the task. The slips-of-action test consisted of six blocks of 24 trials. Response windows and intertrial intervals were both 1 second. The baseline test was identical to the slips-of-action phase except that stimuli were devalued, instead of outcomes, thereby making this a control for general task characteristics, such as having to remember current devaluations and having to refrain from responding. The slips-of-action measure of relative habitual control is a difference score calculated by subtracting the percentage of responses to stimuli with devalued associated outcomes in the slips-of-action test from the percentage of responses to stimuli with associated outcomes that were still valuable. A score of +100 would indicate perfect performance, while a score of zero would indicate a complete failure to selectively withhold responses toward devalued outcomes.

#### Trait control variables

A stop-signal task (SST; Logan, Schachar, & Tannock, 1997) was used to measure inhibitory control, and was included because the ability to inhibit prepotent responses may play an important role in both the slips-of-action test as well as in the switch phase of our experiment in which a well-learnt behavior was disrupted. Higher stop-signal reaction times (SSRT) indicate lesser inhibitory control. As a measure of compulsivity, a Dutch translation of the revised short version of the obsessive-Compulsive Inventory (OCI; Foa et al., 2002) was used. Higher total scores indicate higher compulsivity. As a measure of impulsivity, the Dutch version of the Barrat Impulsiveness Scale (BIS; Bekker, van de Meerendonk, & Mollerus, 2004; Patton et al., 1995; Stanford et al., 2009) was used. The BIS has three subscales: motor impulsivity (BIS-motor), attentional impulsivity (BIS-attention), and nonplanning impulsivity (BIS-planning). Higher total scores indicate higher impulsivity. Further details on these measures can be found in the Supplemental Method section.

#### Exit questionnaire

An exit questionnaire was used to explore several characteristics. It consisted of items concerning strategies used to remember the correct key cover, the extent to which the key-cover procedure and filling in the diary disturbed daily life, the amount of keys on the key set, the amount of other key covers in use, the amount of keys similar to the home key, the percentage of times the home-entry questionnaire was completed on designated days, the degree to which the home-entry questionnaire was completed seriously, the timing of filling out the home entry questionnaire, and the participant’s notion of the purpose of the study.

#### Procedure

**Fig. 1:**
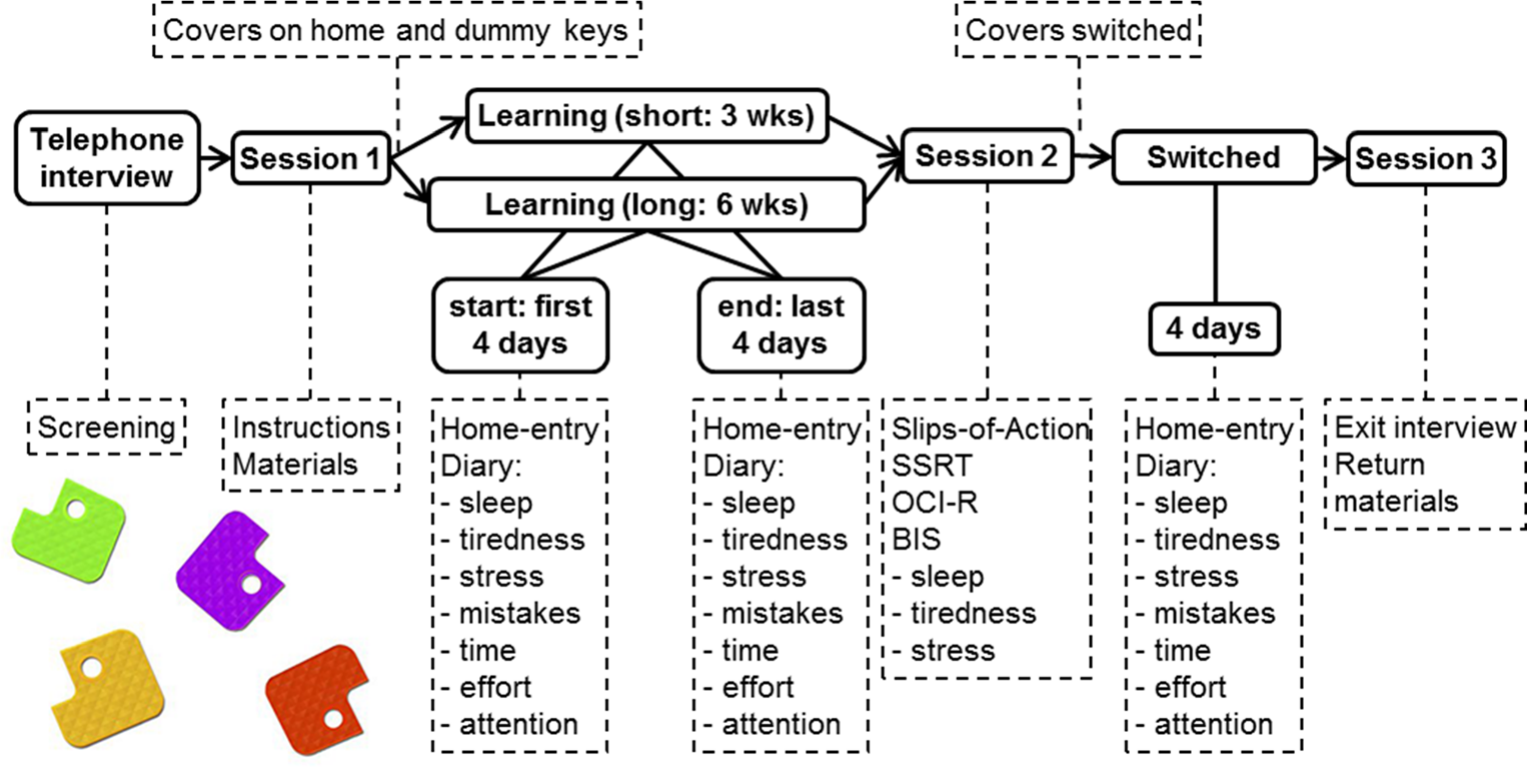
Procedure. Real-life measures were taken during three 4-day periods. Experimental measures were administered in Session 2. This figure also contains examples of the (home and dummy) key covers used in this study

The research was approved by the ethics committee of the University of Amsterdam. An overview of the procedure is given in Fig. 1. Participants were screened in a 10-minute telephone interview. Next, in Session 1, informed consent was obtained, and the PDA, key covers, dummy key, and instructions were supplied. Participants were told that the aim of the research was to investigate the relationship between stress, fatigue, and stability in behavior. Participants were informed that the PDA would record the date and time of each entry. Participants were instructed to report any occasion on which they did not complete the questionnaire within 10 minutes of entering their home. To maximize adherence, participants were asked to describe as precisely as possible where they would place the PDA and how they would incorporate reporting in their home-entry routine. Participants then completed the learning phase of the key-cover procedure. Participants were asked to complete the home-entry diary on every occasion they entered their home in the first and last 4 days of this phase. In Session 2, the slips-of-action task was administered, followed by the first three items of home-entry questionnaire and measures of the following (trait) control variables: inhibitory capacity (SSRT), compulsivity (OCI), and impulsivity (BIS). This session also included instructions for the test phase and lasted approximately 1 hour. The key covers were switched during the first morning of the 4-day test phase. All designated 4-day periods were Mondays through Thursdays. Text messages with a request for confirmation were sent at the start and at the end of each 4-day measurement period. These were also used to indicate the need to start using the key covers or to switch these. In a final 10-minute session, participants completed the exit interview and returned the materials.

## Results

For all analyses, a significance level of *α* = .05 was used and assumptions held, unless otherwise indicated. For pairwise comparisons a Bonferroni correction was applied. All reported correlations are two-tailed and Spearman’s *ρ* is reported where data were not normally distributed.

### Participant characteristics

Forty-two participants were initially admitted to the study. However, two participants dropped out; one participant had incomplete slips-of-action data due to an interrupted task administration, and one participant had incomplete key-cover data by failing to make any diary-entries in the switched phase. Data from these latter two participants were included when analyses did not concern the missing data, and all reported analyses were repeated excluding data from these participants, yielding equivalent results. Data from 38 participants therefore remained for overall analysis. Overall, there were 29 females and nine males, with an average age of 24.4 years (range: 18–39). Chi-square tests and independent-sample *t* tests on all characteristics and control variable scores indicated that the groups (long/short) did not differ significantly. Full descriptive and test statistics of the participant characteristics can be found in online Supplemental Table S1.

### Slips-of-action task

**Fig. 2:**
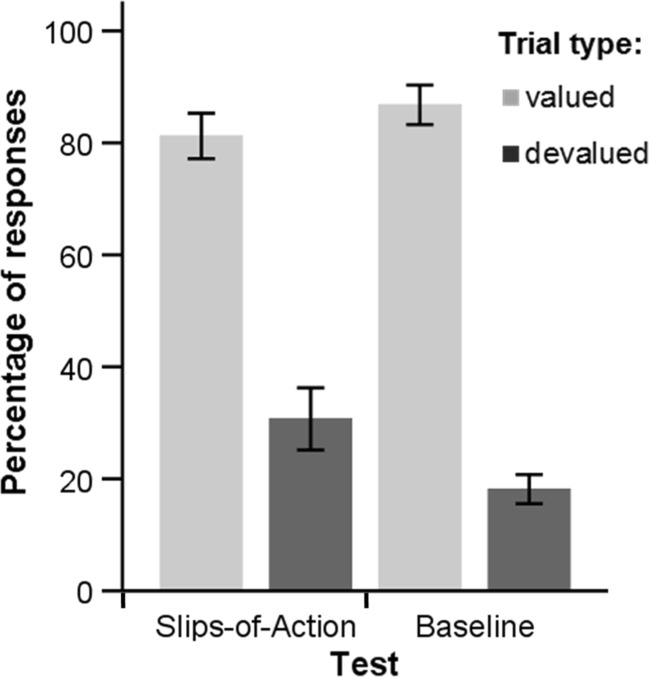
Percentage of responses on valued and devalued trials in the baseline and slips-of-action test. Vertical bars indicate 95% confidence intervals, as calculated using the Loftus and Masson (1994) correction for within-subject designs

Learning in the discrimination-training phase of the slips-of-action task was successful. By the final block, an average accuracy of 92.9% (*SD* = 9.3%) was achieved, which was significantly higher than the 50% chance level, as indicated by a one-sample *t* test, *t*(38) = 28.9, *p* < .001. Figure 2 illustrates performance on the slips-of-action and baseline test.

To investigate performance on both tests, a repeated-measures 2 × 2 ANOVA was conducted, with test (slips-of-action task, baseline) and value (valued, devalued) as within-subject variables, and percentages of responses made as the outcome variable. As expected, overall, participants responded more often on valued trials than on devalued trials, *F*(1, 38) = 330.4, *p <* .001. However, as can be seen in Fig. 2, responding on valued trials and at the same time refraining from responding on devalued trials seems to have been more difficult in the slips-of-action test than in the baseline test. This is consistent with previous studies and was actually predicted, as the baseline test only requires the application of stimulus–response knowledge instead of full stimulus–outcome–response knowledge. Indeed, the interaction effect between value and test was significant *F*(1, 38) = 23.4, *p <* .001. Participants responded less on valued trials in the slips-of-action test (*M* = 81.2%, *SD* = 12.9%) than in the baseline test (*M* = 86.8%, *SD* = 10.9%), *t*(38) = 3.0, *p* = .004 , whereas they responded more on devalued trials in the slips-of-action test (*M* = 30.7%, *SD* = 20.1%) than in the baseline test (*M* = 18.2%, *SD* = 10.1%), *t*(38) = 4.5, *p* < .001. For the subsequent statistical analysis, separate scores on these tests were determined by subtracting the percentage of responses on devalued trials from the percentage of responses on valued trials. On average, participants scored 50.5 (*SD* = 28.7) on the slips-of-action test and 68.6 (*SD* = 17.0) on the baseline test.

### Diary study using the key-cover procedure

#### Diary-study participation

The home-entry diary form was completed 15.7 (*SD* = 4.2) times on average in the 12 designated days, and participants indicated in the exit interview that this represented the majority (*M* = 92%, *SD* = 10.3%) of the actual home entries, although some participants indicated that they completed it less frequently (minimum 51%). The average number of entries in the start-of-learning phase was 6.3 (*SD* = 1.9), which was higher than the average number of entries in both the end-of-learning phase (*M* = 4.6, *SD* = 1.7) and switched phase (*M* = 4.9, *SD* = 1.8), as indicated by paired-sample *t* tests, *t*s(38) = 5.3 and 3.8, *p*s < .001. The lowest number of entries in each 4-day reporting period was two, and the highest was 11. In the exit interview, none of the participants indicated that the task led to a severe disturbance of their daily routine, or to a (correct) idea of what the research was about, nor that it led them to use a particular strategy to remember the correct key cover. Further statistics of measured control variables regarding participation in the diary study can be found in online Supplemental Table S2.

#### Habit formation in the key-cover procedure

**Fig. 3:**
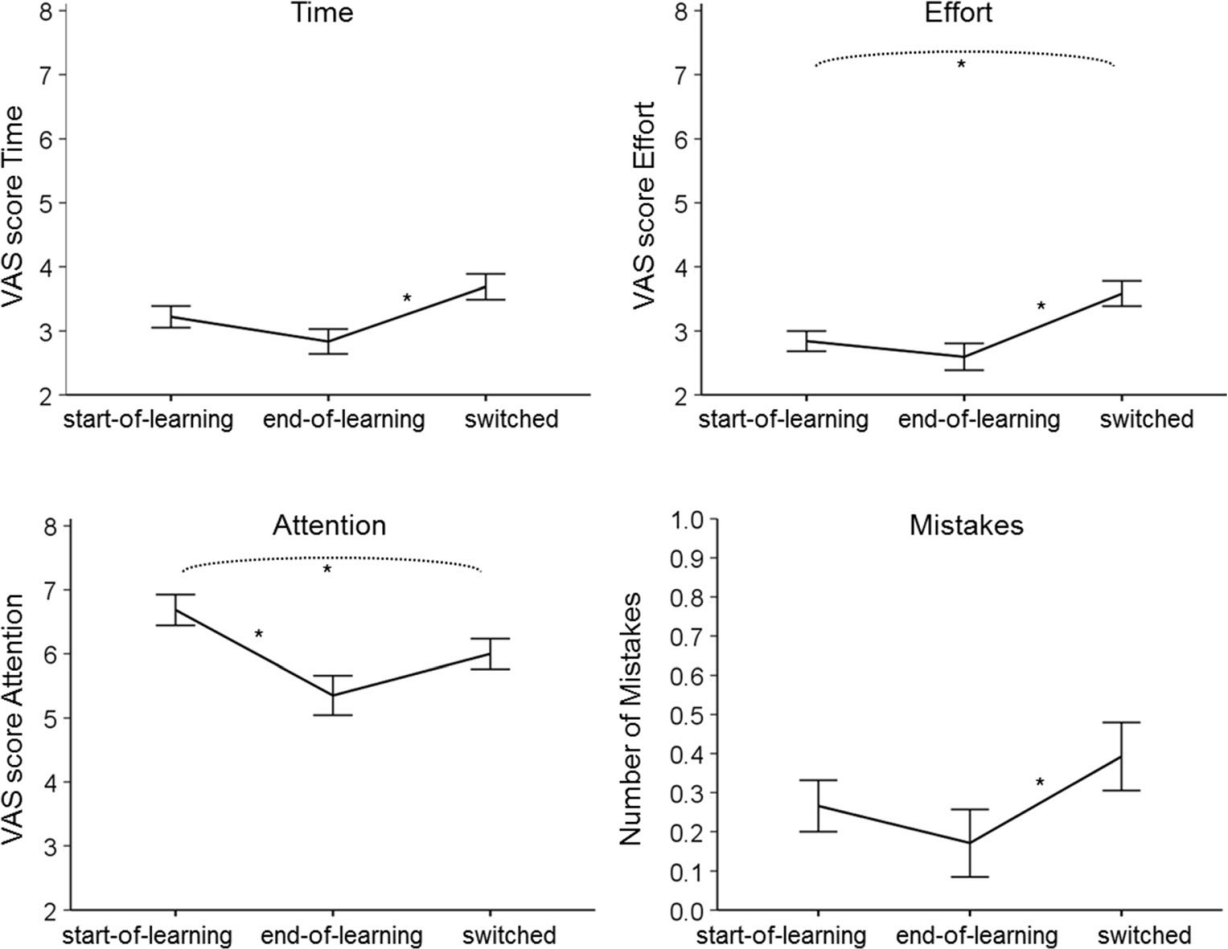
Averages of the automaticity measures in the key-cover procedure across the three 4-day periods. The VAS scores could range between zero and 10. Significant differences are indicated with an asterisk (*). Vertical bars indicate 95% confidence intervals, as calculated using the Loftus and Masson (1994) correction for within-subject designs

Scores on the automaticity measures were first averaged within each 4-day period. The associated descriptive statistics can be found in online Supplemental Table S3. A mixed MANOVA was then conducted, with period (start-of-learning, end-of-learning, switched) as a within-subject variable; group (short, long) as a between-subjects variable; and time, effort, attention and mistakes as outcome variables. Overall, there were significant changes in automaticity over time, *F*(8, 30) = 4.363, *p* = .001. Using Pillai’s trace, there was neither an effect of group, *F*(4, 34) = 1.156, *p* = .347, nor a significant interaction effect, *F*(8, 30) = .735, *p* = .661, indicating that (changes in) automaticity scores were not significantly different in the short and long learning groups. As there were no significant group differences, we concluded that a 3-week learning phase had already produced strong habits. The data were therefore combined in Fig. 3 to show the overall averages of time, effort, attention, and mistakes for each period.

To further investigate the changes in automaticity, separate follow-up ANOVAs were conducted. These revealed that there were significant changes over time for each measure: time, *F*(2, 74) = 6.681, *p* = .002; effort, *F*(2, 74) = 9.651, *p* < .001; attention, *F*(4, 74) = 9.849, *p* < .001; and mistakes, *F*(2, 74) = 5.205, *p* = .008. Figure 3 suggests that all measures showed the same pattern, with first a decrease and then an increase. However, Bonferroni-corrected pairwise comparisons showed that not all individual changes reached significance. From the start-of-learning period to the end-of-learning period, only attention significantly decreased, *p* = .002 (whereas time, effort, and mistakes did not, *p* = .211, *p* = .797, *p* = .632, respectively), thus indicating that one expected benefit of automaticity emerged. From the end-of-learning period to the switched period, time, effort, and mistakes all significantly increased, *p*s = .006, .002, and .048, respectively (whereas attention did not, *p* = .176), thus indicating multiple expected disadvantages of automaticity when situational demands were changed.

Over the entire duration of the procedure (start-of-learning vs. switched period), two opposing patterns can be observed. On the one hand, as expected, resisting automaticity (following the switch, when the key cover cue for the originally learned behavior is still there) requires more effort than the original performance of the key-cover task (before the switch), as effort was significantly higher in the switched period than in the start-of-learning period, *p* = .003. Attention, on the other hand, was significantly lower in the switched period than in the start-of-learning period, *p* = .040, possibly because participation in the study initially generated some additional task awareness. Time and mistakes did not differ significantly in the start-of-learning and switched periods, *p* = .151, *p* = .260.

In summary, behavior in the key-cover procedure—on all automaticity measures—was according to established principles of habit formation and disruption. Therefore, our results support the validity of this paradigm as a model of real-life habit learning.

#### Effects of state variables

To explore the effects of the control state variables (tiredness, stress, and sleep), Spearman correlations with the automaticity measures (time, effort, attention, and mistakes) were calculated. This was done both at the level of the 4-day phases and at the level of the separate PDA entries (unaveraged), as these state variables can be expected to be able to vary from hour to hour as well as from week to week. No significant correlations were found (all *p*s > .05), thus providing evidence against the possibility that changes in automaticity in the present study were due to variability in relevant state variables. It bears mentioning that the assumption of independence of data was not met for the analysis using unaveraged data, as each participant made multiple entries for each measure. It seems likely, however, that participants had relatively stable tendencies across measures to score more or less towards the extremes of the scales. As such, correlation strength was expected to be overestimated in the analysis, rather than underestimated. And therefore, this unmet assumption was presumed not to have masked any possible effect of the state variables.

#### Principal component analysis (PCA)

**Fig. 4:**
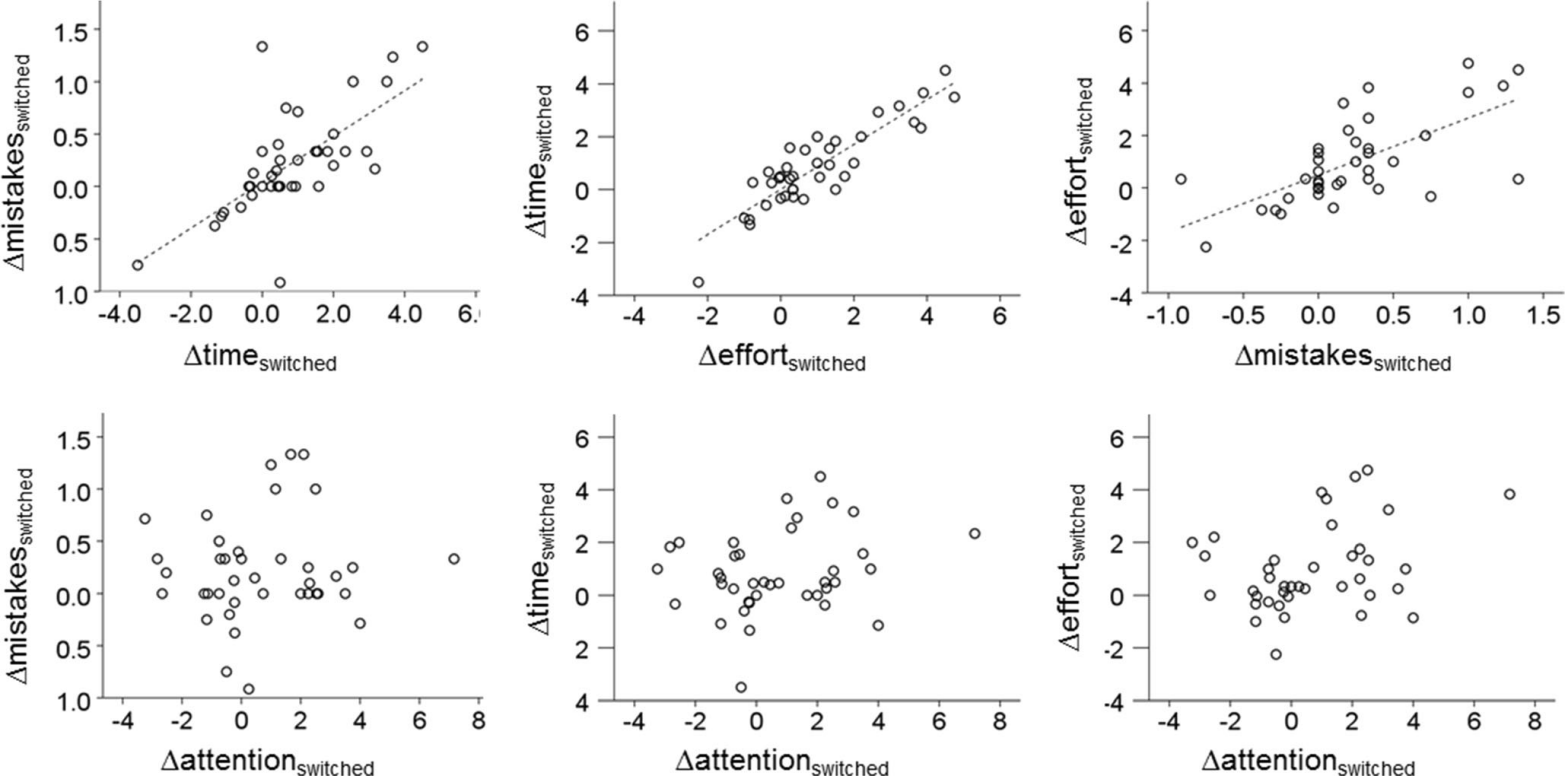
Scatterplots of the automaticity measures of the key-cover procedure in the switched phase. Switched phase difference scores were calculated by subtracting switched scores from end-of-learning scores. Regression lines indicate significant Spearman correlations, all *p*s < .001. Time, effort, and mistakes show linear relations with each other (top row), while attention seems not to correlate with the former measures (bottom row)

Inspection of scatterplots indicated that time, effort, and mistakes had strong linear relationships. For the switched phase, this is illustrated in the top row of Fig. 4. Plotted on the *x*-axis are the switched phase difference scores, which were calculated by subtracting switched scores from end-of-learning scores. For the learning phase, learning phase difference scores were calculated by subtracting end-of-learning scores from start-of-learning scores. Here, results were equivalent. In contrast, attention (bottom row) does not seem to correlate with the other measures.

To further investigate the relationship between time, effort, attention, and mistakes, principal component analyses (PCA) with orthogonal rotation (varimax) were conducted on the switched phase and learning phase difference scores. Two components were extracted in both analyses, as these components had eigenvalues above Jollife’s criterion of .7 (Field, 2009). The eigenvalue of the second component was .93 in the learning phase and .95 in the switched phase. Also, the screen plots showed inflexions at the third component. For the learning phase, these two components together explained 86.9% of the variance. For the switched phase, these two components together explained 88.5% of the variance. Factor loadings after rotation are given in Table 2. Note that very similar patterns occur in the PCAs for both phases.

**Table 2:** Rotated component matrices for both the learning phase and switched phase PCAs (bold font indicates a high factor loading)

Component	Learning phase		Switched phase	
	1	2	1	2
Time	**0.92**	0.16	**0.93**	0.16
Effort	**0.90**	.029	**0.91**	0.27
Mistakes	**0.86**	−0.05	**0.88**	−0.08
Attention	0.11	**0.98**	0.10	**0.99**

As time, effort, and mistakes clustered on one component and attention in itself formed a second one, it seemed that two different aspects of automaticity were measured in the diary study, instead of the expected single construct. The two extracted components for each phase were used in further analyses instead of the separate automaticity measures to avoid the issue of multicollinearity in subsequent regressions.

#### Relation between real-life and experimental habit-formation measures

**Fig. 5:**
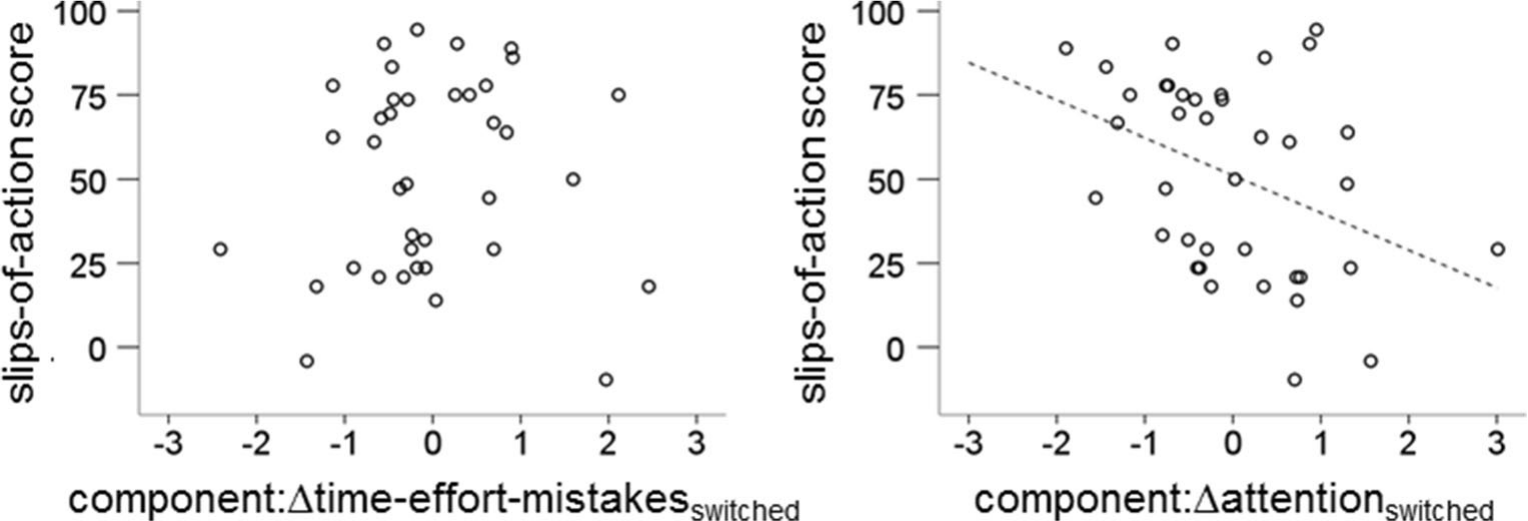
Scatterplots of slips-of-action scores and both components extracted from the switched phase difference scores of the key-cover procedure. The left-hand-side plot shows the behavioral automaticity component and the right-hand-side plot the attentional component. Regression lines indicate significant Spearman correlations, *p*s < .05

In both the slips-of-action task and the switched phase of the key-cover procedure, the habit and goal-directed system are posited to be pitted against each other, and these procedures thereby have been proposed to assess habit propensity. If this is the case, then there should be a strong correlation between their main measures. The scatterplots in Fig. 5 illustrate how slips-of-action performance related to the automaticity components of the key-cover procedure. In the left-hand-side plot, it can be seen how interindividual variability in performance on the slips-of-action task appears to be unrelated to the component mainly based on the time, effort, and mistakes variables. No significant Spearman correlation was found between this behavioral component and vulnerability to slips of action for the switched phase, ρ = .12, *p* = .92. However, in the right-hand-side plot, it can be seen how slips-of-action performance does relate to changes in attention during the key-cover procedure. Specifically, poor slips-of-action performance was related to stronger increases in attention in the switched phase, ρ = −.39, *p* = .016. In contrast, performance on the baseline test did not correlate significantly with automaticity in the key-cover procedure, neither for the behavioral component, ρ = −.01, *p* = .94, nor for the attentional component, ρ = −.09, *p* = .61. This same pattern of results was found when a partial correlation was calculated between slips-of-action performance and attention in the switched phase that controlled for the effect of baseline test performance, ρ = −.41, *p* = .011.

#### Considering trait control variables—Effects of BIS and SSRT

To investigate whether the relationship between slips-of-action task performance, and the attention component of the key-cover procedure was better explained by a third confounding variable, we followed up with a series of regression models. To avoid the risk of overfitting, given the limited number of participants (Field, 2009), one control variable was considered at a time. In each model, the attention component in the switched phase was the predicted variable. The control variables were always entered first as a predictor, followed by slips-of-action scores. The initial regression model was only significant for the control variables BIS-planning and SSRT (*r*s = −.33 and −.44, *p*s = .041 and .006, respectively), when predicting the attention component in the switched phase. But when slips-of-action was entered as the second predictor, the latter was again a significant predictor, leading to significantly better models, *R*^2^ = .244, delta *R*^2^ = .133, *p* = .018, and *R*^2^ = .306, delta *R*^2^ = .112, *p* = .023, respectively. This indicates that although BIS-planning and SSRT could predict some of the variance in attention, a significant part was uniquely predicted by the slips-of-action test. The initial regression model was not significant for any of the 27 other control variables (see online Supplemental Table S2 for complete listing). It was concluded that the relationship between slips-of-action task performance and the attention component of the key-cover procedure was not better explained by any control variable. Moreover, none of the state variables and variables possibly associated with repetition, behavior novelty, and motivation could significantly predict the attention component in the key-cover procedure.

Although the time-effort-mistakes component of the key-cover procedure was not significantly related to slips-of-action performance, control regressions were identically constructed to explore whether suppressor effects (Smith, Ager, & Williams, 1992) could explain the mentioned lack of correlation, or if any of the other abovementioned control variables could predict this component. Except for the amount of other key covers already in use by participants, none of these could significantly predict the outcome. The more other key covers were in use, the stronger was the increase in effort, time, and mistakes in the switched phase, *F*(1, 36) = 8.79, *p* = .005, *R*^2^ = .196, with standardized beta = .44. When slips-of-action was entered as the second predictor in all these models, it remained unrelated to the time-effort-mistakes component of the key-cover procedure. In total, eight participants had at least one other key cover in use. To make sure these had not influenced the overall results, all analyses mentioned in this section were repeated excluding these participants, with equivalent and equally significant results. It was concluded that no control variables could predict the variance in the time-effort-mistakes component of the key-cover procedure, nor could they explain its lack of correlation with slips-of-action performance.

## Discussion

Are some people inherently more “a creature of habit”’ than others? If so, then individual differences in habit propensity should be observable across different domains. The present study therefore set out to investigate individual dispositions in the balance between goal-directed and habitual control in real life, as well as in experimental settings. This was achieved by relating interindividual differences in habitual control as measured by an experimental paradigm—the slips-of-action task—to automaticity of a real-life behavior, namely, selecting a key to open the front door of one’s home whilst controlling both habit formation and habit disruption experiences. If people indeed have differential habit propensity, then these experimental and real-life measures of habit propensity should be directly related to each other. This hypothesis—fitting with the idea of habit propensity as a relatively stable personal characteristic—did not receive strong support from the current study. In short, we found that focused attention—as a measure of automaticity—correlated significantly with performance on the experimental slips-of-action task. This association could not be explained by any control variables. However, no such relationship was found between task performance and other real-life measures of automaticity, namely, time, effort, and mistakes.

Overall, the data from the diary study were in line with the established properties of habits. There were indications of the benefits of increasing efficiency with repetition during the learning phase, as reflected in a decrease in the attention required to select the correct key. Also, when the habit was disrupted during the switched phase, the downside of inflexibility emerged, as indicated by increases in effort, time, and mistakes. Effort even increased beyond its initial level. Against our expectation that all four automaticity measures would reflect a single automaticity construct, we found evidence for two components. One component concerned the degree to which key selection was the focus of attention, whilst the other concerned the time, effort, and mistakes involved in the key-selection process. Possibly this latter component better reflects automaticity in the sense of learning a motor skill, as a consequence of stimulus–response learning (Dezfouli & Balleine, 2012; Doyon & Benali, 2005). Note that the finding of two such distinct components does not seem to be at odds with findings in previous diary studies into habits, where no reports were made on the possible intercorrelation of the different indicators of automaticity (Wood et al., 2002).

As previously mentioned, when relating these two components to performance on the experimental measure of habit propensity, only for attentional automaticity was the expected correlation with slips-of-action performance found. To clarify this relationship, we propose that increased attention after the key-cover switch indicates a need to recruit more cognitive resources as a consequence of a relatively ineffective goal-directed system. In other words, people with poor goal-directed control are consequently relatively dependent on S-R habits, and may therefore require more attentional control to compensate for a lack of flexibility. A finding supporting this post-hoc interpretation is that attention in the key-cover procedure also corresponded negatively with inhibitory capacity as measured with the Stop Signal Task. This means that people with weaker inhibitory capacity require more focused attention on the task in order to allow for flexible behavioral adjustment.

The question remains why focused attention correlated with slips-of-action performance whereas the ‘motor automaticity’ component did not. We suggest that the specific properties of the slips-of-action task and the key-cover procedure influence the relative contributions of the goal-directed and habitual system to action control. The slips-of-action task on the one hand, is plausibly more sensitive to the strength of the goal-directed system than to the strength of the habit system, because of the relatively limited amount of instrumental training that participants receive in this and other studies. Stimulus-response links are not likely to be over-trained yet and outcome knowledge may still be imperfect. This assumption is supported by the correlation (*r* = .61) between response-outcome knowledge and the slips-of-action task performance found by Gillan et al. (2011). Also, de Wit, Watson, et al. (2012b) found that the integrity of the brain network associated with the goal-directed system explained more variance in slips-of-action scores (*r*^*2*^ = .50 ) than the integrity of the brain network associated with the habit system did (*r*^*2*^ = .23 to *r*^*2*^ = .34). Furthermore, in a recent study, model-based control during the two-step task showed a moderately positive correlation with performance on the slips-of-action task, whereas model-free control did not (Sjoerds et al., 2016). Similarly, the very nature of goal-directed action presupposes attentional engagement (Wood & Neal, 2007) and therefore, attention—as measured during the key-cover procedure—also might be more reflective of the strength of the goal-directed system. In short, both attention and the slips-of-action performance may well be more sensitive to strength and activity of the goal-directed system, thus explaining the found association between these measures. The motor automaticity component on the other hand—as indicated by changes in reported effort, time needed, and mistakes made during the key-cover procedure—may be more sensitive to the strength of the habit system, thus explaining its lack of association with slips-of-action task performance. Key selection only involved a single stimulus–outcome–response relation, that was apparently remembered easily by participants, as in the exit interview several participants spontaneously mentioned that they memorized the correct key color right away. Therefore the goal-directed system’s capacity for acquiring (declarative) outcome knowledge may not have been as much of a bottleneck as it is in the slips-of-action task. The habit system’s capacity for quickly forming strong stimulus–response associations that autonomously drive behavior is more likely to be essential for mistakes as well as increased effort and time induced by the key-cover switch.

Having said this, two separate (sMRI) imaging studies do offer some tentative, indirect evidence that this paradigm captures to some extent the contributions of the habit system (de Wit et al., 2012a; Delorme et al., 2016). Therefore, the possibility that the presently administered version of the slips-of-action task is relatively sensitive to the strength of the goal-directed system should be further investigated in future research. We predict that if a longer training phase is used in the slips-of-action task, its sensitivity for the strength of the habit system should increase. Consequently, an association with motor automaticity during the key-cover procedure may emerge. It remains an important challenge for our research field to develop an experimental paradigm that is sufficiently sensitive to reveal both the contributions of the goal-directed and habitual system to the flexibility and efficiency of action control (Watson & de Wit, 2018). At present, it appears as if existing tasks—slips-of-action task, the avoidance habit task, and the two-step task—are all relatively sensitive to functioning of the goal-directed system (de Wit, Watson, et al., 2012b; Friedel et al., 2014; Gillan et al., 2014a, 2015b; Sjoerds et al., 2016).

A key contribution of the current research is the novel key-cover procedure. It allows real-life investigations of habit propensity, as factors that would normally confound any naturalistic investigation into individual differences in habit propensity are controlled for. First, a stable cue is provided by the arrival at the front door. Second, the amount of motivation to select the right key can be expected to be similar: Every participant wants to open the door and go inside. Third, task novelty is ensured by supplying new key covers. This is supported by the finding that previous duration of key use was not related to automaticity. Finally, the amount of behavior repetition is determined by the study length as variations in the amount of home entries per day seem sufficiently low, and the number of home entries was not associated with automaticity.

Although behavior during the key-cover procedure was generally in line with the established principles of habit formation, some expected changes in automaticity measures did not reach significance. We suspect that the learning phase, by not yet provoking a clear response conflict, may not elicit many mistakes or changes in effort and time, thus explaining why decreases of these measures were not significant in this relatively small sample. Attention, however, may have initially been elevated because of some additional task awareness generated by the novelty of study participation. This effect may have been greatest in the first few days, and it may have further diminished over the course of the study, thus explaining why decreases in attention were significant in the learning phase, whereas increases in the switched phase were not. In support of this idea of additional awareness due to study participation, we found that significantly more entries were made during the first days of the learning phase. This additional awareness might be a downside of the diary-study approach. However, given the significant correlation between attention and the experimental slips-of-action task, this seems not to have influenced the key-selection process too much.

The diary-study approach that was taken has certain advantages over a questionnaire approach. First, behavior is recorded directly after it occurred, such that recall problems that can occur using the SRHI (Gardner & Tang, 2014), or any other delayed questionnaire, are not an issue. Specifically, for habitual responses that are performed with little attention, memory for details such as the amount of effort and time needed might well be unreliable. Second, the diary-study approach allowed us to investigate inflexibility very specifically by probing actual mistakes made in the key-selection process instead of relying on items subjectively gauging how much control one has over a particular behavior. By thus facilitating the naturalistic investigation of habit propensity, diary studies using the key-cover procedure open up a new area of investigation in the research program into habit formation. Indeed, we regard the development of this well-controlled procedure to study habit formation in a real-word setting as the main contribution of the current study.

Our finding that focused attention in the key cover paradigm correlated significantly with performance on the experimental slips-of-action task is in line with habit propensity as a relatively stable personal characteristic. However, until these results are replicated, they should be treated with caution. Furthermore, alternative approaches to address this question should be explored. An important outstanding question for future research is whether performance on habit measures shows stability over time. Future studies could achieve this by assessing individuals’ performance across long intervals. Similarly, the stability of the associated brain circuit integrity should be investigated, both after the simple passage of time and after events such as successful therapy for disorders associated with habit propensity. That being said, we already have tentative evidence from previous studies that performance on the slips-of-action paradigm does not remain stable across the life span. Healthy aging has been associated with impaired performance (de Wit, Vijver, & Ridderinkhof, 2014; and see Eppinger, Walter, Heekeren, & Li, 2013, for related evidence using a computational modeling approach), which may be due to age-related changes in brain functioning. Finally, we also know that dietary manipulations that affect dopamine and serotonin function can acutely affect people’s vulnerability to “slips of action” (de Wit, Standing. et al., 201; Worbe et al., 2015). These findings notwithstanding, the notion that habit propensity is a personal characteristic with relative stability would imply that habitual propensity is at least preserved to some degree over time and context.

Considering that habitual behavior is implicated in important domains such as health behaviors (Aarts & Dijksterhuis, 2000; Danner, Aarts, Papies, & de Vries, 2011; Lally & Gardner, 2013; Quinn, Pascoe, Wood, & Neal, 2010), addiction (Ersche et al., 2016; Everitt, Dickinson, & Robbins, 2001; Everitt & Robbins, 2005; Sjoerds et al., 2013), eating disorders (Graybiel, 2008), and obsessive- compulsive disorder (Gillan et al., 2011, 2014b, 2015a), we believe that habit propensity could be an important concept. Furthermore, habit propensity may significantly influence the best strategy to modify one’s behavior, either by focusing on goals and motivation (Miller & Rollnick, 2009), or, for example, on retraining automatic tendencies (Wiers, Eberl, Rinck, Becker, & Lindenmeyer, 2011). Relatedly, habit propensity may moderate the effectiveness of implementation intentions (Belanger-Gravel, Godin, & Amireault, 2013). In a clinical setting, the question emerges as to whether people with a strong habit propensity benefit more from interventions such as habit reversal (Dutta & Cavanna, 2013) than from interventions that offer “mere” exposure with response prevention (van de Griendt, Verdellen, van Dijk, & Verbraak, 2013). Clearly, these speculations warrant future investigation.

To summarize, in the present study, we have introduced a novel procedure that allows the investigation of habit propensity in a real-life setting, and we present some initial evidence supporting the idea that this is a broad trait-like characteristic that influences a wide range of behaviors. We hope that the approach taken in the present study will inspire further research into the potentially important concept of habit propensity.

## Electronic supplementary material

ESM 1 (DOCX 34 kb)
