# Supplementary material for: Investigating the balance between goal-directed and habitual control in experimental and real-life settings
Source: Learn Behav. 2018 Feb 9;46(3):306–19. doi: 10.3758/s13420-018-0313-6 (PMC6105188; doi:10.3758/s13420-018-0313-6)
Supplement: Supplementary file 1 — (DOCX 34 kb) [file 13420_2018_313_MOESM1_ESM.docx]

**Supplemental Method**

Contains: Additional details on methods (BIS, OCI, SST), additional descriptive and test statistics on participation and performance in the diary study using the key-cover procedure, full listing of control variables used.

**Stop Signal Task**

A Stop Signal Task (SST; Logan et al., 1997) was used to measure inhibitory control. The specific version of the task used colored visual go signals appearing on the right and left of the screen, requiring right and left button presses from the participants. Colored visual stop signals were presented on 25% of the trials and required the inhibition of the button press responses. Stop signal timing was dynamically adjusted to allow successful response inhibition for 50% of the trials. This 50% success rate was confirmed using a one sample T-test. Three learning blocks with a total of 48 trials were followed by two testing blocks with a total of 168 trials. Both verbal and written instructions were provided. The stop signal reaction time (**SSRT**) was calculated by subtracting the mean stop-signal delay from the mean go-signal reaction time. Higher SSRT times indicate lesser inhibitory control.

**Obsessive-Compulsive Inventory (OCI)**

As a measure of compulsivity, the Dutch version (Droogendijk & Eeuwijk van, 2011) of the revised short version of the Obsessive–Compulsive Inventory (**OCI**; Foa et al., 2002) was used. Items include statements such as: “I find it difficult to control my own thoughts” and “I need things to be arranged in a particular order”, which are rated on a five point scale with the range: 0 - “not at all”, 1 - “a little”, 2 - “moderately”, 3 - “a lot”, and 4 - “extremely”. Scores are summed and higher total scores indicate higher compulsivity. The OCI-R has adequate test-rest reliability and shows good convergent and divergent validity (Hajcak, Huppert, Simons, & Foa, 2004)

**Barratt Impulsiveness Scale (BIS)**

As a measure of impulsivity, the Dutch version (Bekker et al., 2004) of the Barrat Impulsiveness Scale (**BIS**-11;Patton et al., 1995) was used. Items include statements such as: “I do things without thinking” and “I plan tasks carefully”, which are rated on a four point scale with the range: 1 - “never / rarely”, 2 - “occasionally”, 3 - “often”, and 4 - “almost always / always”. Some items such as the second example are reverse scored. Higher total scores indicate higher impulsivity. The BIS-11 has three subscales: motor impulsivity, attentional impulsivity, non-planning impulsivity. The BIS-11 has a good internal consistency across populations and has good convergent validity with other self-report measures (Stanford et al., 2009).

**Supplemental Results**

**Supplemental Table 1. Participation characteristics**

*descriptive and test statistics*

|  | short learning phase (*n*=20) | | | |  | long learning phase (*n*=18) | | | |  | group equality | |
| --- | --- | --- | --- | --- | --- | --- | --- | --- | --- | --- | --- | --- |
|  | *Mean* | *SD* | *Min* | *Max* |  | *Mean* | *SD* | *Min* | *Max* |  | *T* | *p* |
| age | 23.8 | (4.9) | 18 | 33 |  | 25 | (5.9) | 19 | 39 |  | -.8 | .44 |
| BIS | 63.2 | (7.5) | 50 | 79 |  | 65 | (7.4) | 45 | 78 |  | -.7 | .48 |
| OCI | 11.8 | (8.0) | 2 | 30 |  | 15 | (8.7) | 3 | 35 |  | -1.3 | .21 |
| SSRT | 88 | 60 | 13 | 251 |  | 64 | 48 | -5 | 188 |  | 1.3 | .19 |
| start-of-learning entries | 6.0 | (1.5) | 3 | 8 |  | 6.7 | (2.3) | 3 | 11 |  | -1.1 | .30 |
| end-of-learning entries | 4.9 | (1.9) | 2 | 9 |  | 4.4 | (1.4) | 2 | 8 |  | .8 | .41 |
| switched entries | 4.9 | (2.0) | 2 | 9 |  | 5.0 | (1.7) | 2 | 9 |  | -.3 | .80 |
| total entries | 15.7 | (4.3) | 8 | 24 |  | 16.1 | (4.2) | 9 | 25 |  | -.3 | .80 |
| average tiredness (1 - 10) | 5.1 | (0.9) | 3.1 | 6.4 |  | 5.6 | (0.8) | 4.1 | 7.4 |  | -1.7 | .09 |
| average sleep | 7.4 | (0.7) | 5.9 | 8.7 |  | 7.7 | (0.7) | 6.6 | 9.4 |  | -1.3 | .22 |
| average stress (1 - 10) | 3.8 | (0.8) | 2.1 | 5.1 |  | 3.7 | (0.6) | 2.9 | 5.2 |  | .2 | .83 |
| number of keys on key-set | 4.9 | (2.7) | 0 | 10 |  | 5.5 | (2.4) | 2 | 11 |  | -.7 | .48 |
| other key-covers on key-set | 0.4 | (0.8) | 0 | 3 |  | 0.3 | (0.6) | 0 | 2 |  | .3 | .76 |
| home key usage (months) | 31.0 | (35.4) | 1 | 108 |  | 39.1 | (45.3) | 2 | 180 |  | -.6 | .54 |
| PDA completion (0 - 100%) | 93.5 | (8.1) | 74 | 99 |  | 91.4 | (11.5) | 51 | 99 |  | .6 | .52 |
| seriousness (0 - 100%) | 90.8 | (11.8) | 57 | 99 |  | 91.7 | (10.6) | 59 | 99 |  | -.3 | .80 |

**Supplemental Table 2. Diary study participation**

|  |  | total, *N* = 39 | | | |  | short, *n* = 20 | | | |  | long, *n* = 19 | | | |
| --- | --- | --- | --- | --- | --- | --- | --- | --- | --- | --- | --- | --- | --- | --- | --- |
|  |  | *Mean* | *SD* | *Min* | *Max* |  | *Mean* | *SD* | *Min* | *Max* |  | *Mean* | *SD* | *Min* | *Max* |
| sleep | start-of-learning | 7.5 | (0.7) | 6.0 | 9.4 |  | 7.3 | (0.7) | 6.0 | 8.4 |  | 7.7 | (0.7) | 6.3 | 9.4 |
|  | end-of-learning | 7.5 | (0.9) | 5.5 | 10.3 |  | 7.6 | (0.9) | 6.3 | 10.3 |  | 7.5 | (0.9) | 5.5 | 9.3 |
|  | switched | 7.6 | (0.7) | 6.0 | 9.7 |  | 7.4 | (0.7) | 6.0 | 8.5 |  | 7.8 | (0.7) | 6.8 | 9.7 |
|  |  |  |  |  |  |  |  |  |  |  |  |  |  |  |  |
| tiredness | start-of-learning | 5.5 | (1.2) | 3.1 | 7.8 |  | 5.2 | (1.2) | 3.1 | 7.7 |  | 5.8 | (1.2) | 3.3 | 7.8 |
|  | end-of-learning | 5.3 | (1.6) | 2.4 | 8.3 |  | 4.9 | (1.5) | 2.4 | 7.4 |  | 5.7 | (1.5) | 3.3 | 8.3 |
|  | switched | 5.5 | (1.1) | 2.8 | 8.3 |  | 5.4 | (1.2) | 3.2 | 8.3 |  | 5.7 | (1.1) | 2.8 | 7.3 |
|  |  |  |  |  |  |  |  |  |  |  |  |  |  |  |  |
| stress | start-of-learning | 3.5 | (1.1) | 1.0 | 5.5 |  | 3.7 | (1.0) | 1.6 | 4.8 |  | 3.3 | (1.2) | 1.0 | 5.5 |
|  | end-of-learning | 3.5 | (1.4) | 1.0 | 6.5 |  | 3.3 | (1.1) | 1.3 | 4.8 |  | 3.7 | (1.6) | 1.0 | 6.5 |
|  | switched | 3.6 | (1.2) | 1.4 | 6.0 |  | 3.7 | (1.2) | 1.4 | 6.0 |  | 3.4 | (1.1) | 1.8 | 5.0 |

*Descriptive statistics of main state control variables.* **The complete list of 29 control variables that were used in the series of regression models contained: OCI, BIS, BIS-motor, BIS-Planning, BIS-attention, SSRT, education, age, baseline-test performance, learning -phase stress, session-2 stress, switched-phase stress, learning-phase tiredness, session-2 tiredness, switched-phase tiredness, learning-phase sleep, session-2 sleep, switched-phase sleep, number of start-of-learning entries, number of end-of-learning entries, number of switched entries, total number of entries, reward type, reported seriousness of participation, reported percentage of PDA completion, number of keys on the key-set, number of months living in the house, number of similar keys, and number of other key-covers in use.*

**Supplemental Table 3. Effects of the key-cover procedure**

*Descriptive statistics of automaticity measures*

|  |  | short, *n* = 20 | | | |  | long, *n* = 19 | | | |
| --- | --- | --- | --- | --- | --- | --- | --- | --- | --- | --- |
|  |  | *Mean* | *SD* | *Min* | *Max* |  | *Mean* | *SD* | *Min* | *Max* |
| effort | start-of-learning | 2.9 | (1.2) | 1 | 5 |  | 2.8 | (1.1) | 1 | 5 |
|  | end-of-learning | 2.8 | (1.6) | 1 | 6 |  | 2.3 | (1.1) | 1 | 6 |
|  | switched | 3.6 | (1.4) | 1 | 6 |  | 3.5 | (1.5) | 2 | 7 |
|  |  |  |  |  |  |  |  |  |  |  |
| time | start-of-learning | 3.2 | (1.4) | 1 | 6 |  | 3.2 | (1.3) | 1 | 6 |
|  | end-of-learning | 3.1 | (1.4) | 1 | 6 |  | 2.5 | (1.2) | 1 | 6 |
|  | switched | 3.8 | (1.4) | 1 | 6 |  | 3.5 | (1.3) | 1 | 6 |
|  |  |  |  |  |  |  |  |  |  |  |
| attention | start-of-learning | 7.0 | (1.3) | 5 | 10 |  | 6.4 | (1.9) | 2 | 9 |
|  | end-of-learning | 5.8 | (2.0) | 2 | 10 |  | 4.7 | (2.5) | 1 | 9 |
|  | switched | 5.9 | (1.5) | 4 | 10 |  | 6.0 | (1.3) | 4 | 9 |
|  |  |  |  |  |  |  |  |  |  |  |
| mistakes | start-of-learning | .30 | (.24) | 0 | .7 |  | .25 | (.26) | 0 | .8 |
|  | end-of-learning | .25 | (.37) | 0 | 1.3 |  | .11 | (.22) | 0 | .8 |
|  | switched | .42 | (.35) | 0 | 1.0 |  | .38 | (.50) | 0 | 1.4 |
